# Antiherpetic Activity of Carrageenan Complex with Echinochrome A and Its Liposomal Form

**DOI:** 10.3390/ijms232415754

**Published:** 2022-12-12

**Authors:** Natalia V. Krylova, Vladimir I. Gorbach, Olga V. Iunikhina, Anastasia B. Pott, Valery P. Glazunov, Anna O. Kravchenko, Mikhail Y. Shchelkanov, Irina M. Yermak

**Affiliations:** 1 G.P. Somov Institute of Epidemiology and Microbiology, Rospotrebnadzor, 690087 Vladivostok, Russia; 2 G.B. Elyakov Pacific Institute of Bioorganic Chemistry, Far-Eastern Branch of the Russian Academy of Sciences, 690022 Vladivostok, Russia

**Keywords:** carrageenan complex, herpes simplex virus type 1, echinochrome A, liposome

## Abstract

Herpes simplex virus (HSV) infections, the incidence of which is still widespread throughout the world, are actualizing the search and development of new, more effective antiherpetic drugs. The development of multifunctional drug delivery systems, including liposome-based ones, has become a relevant and attractive concept in nanotechnology. The ability of complexes of κ- and Σ-carrageenans (CRGs)—sulfated polysaccharides of red algae, with echinochrome A (Ech), as well as the liposomal form of the Σ-CRG/Ech complex—to inhibit different stages of HSV-1 infection in Vero cells was studied. By quantum chemical calculations, it was shown that CRG forms stable complexes with Ech. We have shown that complexes of κ-CRG/Ech and Σ-CRG/Ech exhibit highest virucidal activity with a selectivity index (SI) of 270 and 350, respectively, and inhibition of virus-cell interaction (SI of 83 and 32, respectively). The liposomal form of the Σ-CRG/Ech complex after virus adsorption and penetration to cells effectively reduced the HSV-1 plaque formation. The virus-inhibiting activity of the liposomal form of the Σ-CRG/Ech complex was three times higher than that of the Σ-CRG/Ech complex itself. Obtaining CRGs/Ech complexes and their liposomal forms can become the basis of a successful strategy for the development of promising antiherpetic drugs.

## 1. Introduction

Herpes simplex virus type 1 (HSV-1), a member of the *Herpesviridae* family, remains one of the most common human viral pathogens [1]. The worldwide prevalence of HSV-1 is largely due to the fact that it establishes a lifelong latent infection and can periodically reactivate to cause disease [2]. Recently, the number of reports on the resistance of HSV-1 against many drugs based on nucleoside analogues, which are acyclovir and its derivatives, has increased [3,4,5]. Therefore, the search for new antiviral compounds that differ from acyclovir and its analogues in the mechanism of action by selectively inhibiting the processes of adsorption and reproduction of the virus, without harming the host organism, and combining antiviral, anti-inflammatory, and immunomodulatory properties is one of the urgent tasks of medical virology.

According to the generalized literature data, sulphated polysaccharides (SPs) of seaweeds are potential antivirals that can interfere with the early stages of viral replication, including the virus entry. Due to the presence of a negative charge on their surface, SPs mimic heparin sulfate residues present on the proteoglycans on the surface of target cells. SPs mask the positive charge of virus surface receptors and therefore prevent their binding to host-cell proteoglycans [6]. Sulphated polysaccharides of red algae, carrageenans (CRGs), exhibit a wide spectrum of biological activity and have been effective mainly against enveloped viruses [7,8,9]. Today, CRGs, due to the safety of the application (included in (USP35-NF30S1), (BP2012), and (EP7.0)), have a wide range of applications in the biomedical field as an excipient. CRGs are sulphated linear galactans, whose basic structural units are a disaccharide, carrabiose, consisting of alternating β-1,3 (G-units) and α-1,4 (D-units)-linked galactose residues. The 1,4-linked residues are commonly, but not invariably, present as 3,6-anhydro-α-D-galactopyranose (DA-units) [10,11]. Among various types of CRGs, there are three major types that are commercially available, which are designated as kappa (G4S-DA), iota (G4S-DA2S), and lambda (G2S-D2S, 6S) [12].

Earlier, we showed that CRGs had antiviral activity against both DNA-containing HSV-1 and RNA virus (enterovirus ECHO-1) by significantly increasing the cell resistance to virus infection and preventing the attachment and penetration of virus to cells [13]. We investigated different types of CRGs and detected that their antiviral activity depends on the CRGs’ structure. CRGs are currently being investigated as a drug delivery matrix [14,15]. We have shown that CRGs, isolated from Pacific red alga, have mucoadhesive properties [16] that may be useful for topical and oral drug delivery [17]. Earlier, we showed that CRG improved the solubility of a water-insoluble red pigment of sea urchins, echinochrome (7-ethyl-2,3,5,6,8-pentahydroxy-1,4-naphthoquinone, Ech) [18], and also prevented its oxidative degradation. Moreover, it has been previously shown that Ech and its derivatives exhibit antiviral properties [19,20]. The ability of CRGs to dissolve and protect Ech from oxidation, as well as the antiviral activity of these substances, opens up the possibility of creating composites based on them with enhanced antiviral activity. In this work, quantum chemical calculations were used to prove the formation of CRG/Ech complex and to visualize and identify possible binding sites.

Carbohydrate-based liposome vehicles with the capability of reducing dosing frequency, improving drug pharmacological activity, and delivering drugs at the specified site appear to be promising as pharmaceutical drug carriers [21]. The inclusion of drugs in liposomes offers the potential for localized and sustained delivery to mucosal [22,23]. Liposomal drugs have been successfully used in various fields of medicine for external and systemic use. Prescribing liposomes as drug delivery systems for treating infectious diseases and tumors has been well documented [24]. The incorporation of a hydrophobic Ech into liposomes has been previously shown by us [25].

The aim of this work was to evaluate the complex action of CRG and Ech on the herpes simplex virus type 1. In this experimental work, we also obtained liposomes containing CRG/Ech complex and evaluated their anti-HSV-1 effect.

## 2. Results 

### 2.1. Chemical Structures of the CRGs and Ech 

The CRGs were isolated from red seaweed *Chondrus armatus* (*Gigartinaceae*) by hot water, filtered through a Vivaflow200 membrane for purified from low-molecular-weight impurities, and precipitated from solutions by alcohol. 

The yield of total polysaccharide fraction from seaweed (Σ-CRG) was 50%. The Σ-CRGC was separated into the gelling and the nongelling fractions after 4% KCl precipitation, as described in [26]. According to the chemical analysis, Σ-CRGC and its fractions consisted of galactose (Gal) and 3,6-fyhydrogalactose (3,6 AnGal). The structures of the disaccharide repeating units of the CRGs’ fractions and viscosimetric molecular weights of CRGs are listed in Table 1. 

FTIR and NMR spectra were used to identify the polysaccharide structure of the gelling fraction, and the obtained spectra were compared with the spectra of the polysaccharides isolated from *C. armatus* by us earlier [26].

Absorption bands in the IR spectra and chemical shifts in the NMR spectra were assigned via comparison to signals of known carrageenan structures [10,27].

**Figure 1:**
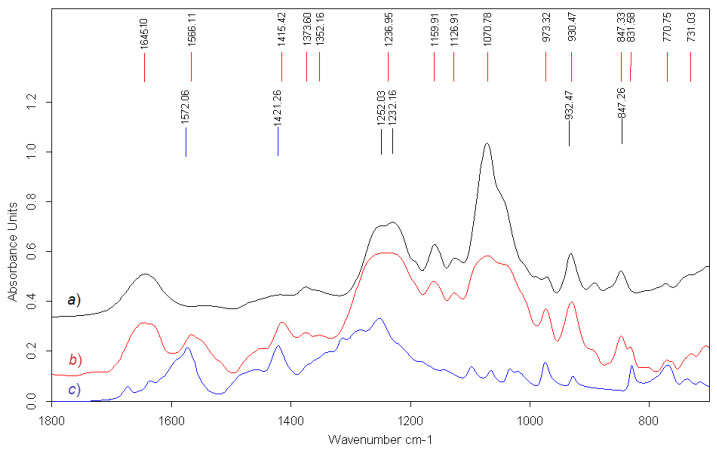
FTIR spectra: (**a**) κ-CRG, (**b**) κ-CRG/Ech, and (**c**) Ech.

The IR spectra of Σ-CRG and gelling polysaccharide showed a poor resolved absorption strong band with maxima at 1252 and 1232 cm^−1^ (Figure 1a), as is characteristic of sulfate groups (asymmetric stretching vibrations S=O bonds of SO3 group) and in agreement with the results of the chemical analysis (Table 1). This spectrum showed absorption bands at 930 cm^−1^ for 3,6-anhydrogalactose (C(3)–O–C(6) stretching vibration) and the second stretching vibration S–O bonds of 847 cm^−1^ characteristic of the axial sulfate group at C-4 of the 3-linked β-D-galactose. Our analysis of the FTIR spectra of the gelling polysaccharide confirmed that it was mainly κ-CRG.

**Figure 2:**
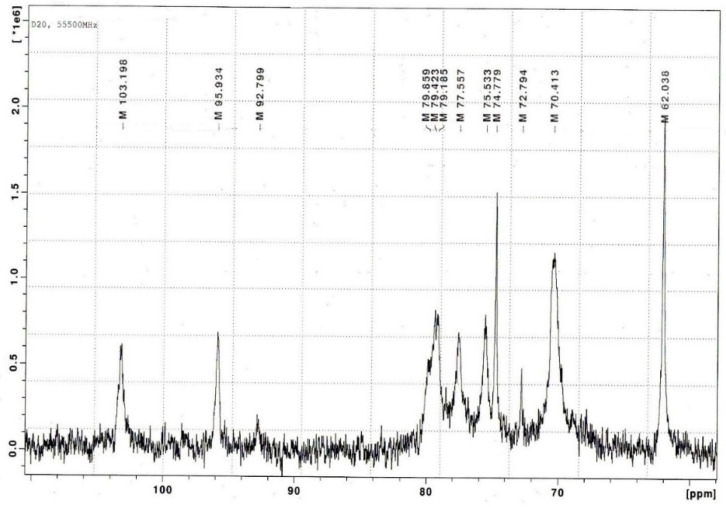
^13^C-NMR- spectrum of κ-CRG from *C. armatus*.

The FTIR data were confirmed by NMR spectroscopy (^1^H and ^13^C NMR analyses). The two signals at 103.1 ppm and 95.9 ppm in the anomeric carbon resonance area of the ^13^C-NMR spectra of gelling polysaccharide from *C. armatus* were assigned to C-1 of the three-linked β-D-galactose residue (G4S) and C-1 of the four-linked 3,6-anhydro-α-D-galactose (DA) of κ-CRG (Figure 2). 

The unfractionated Σ-CRG consisting of κ and λ-CRGs with the ratio 60:40 [26] was used also.

**Figure 3:**
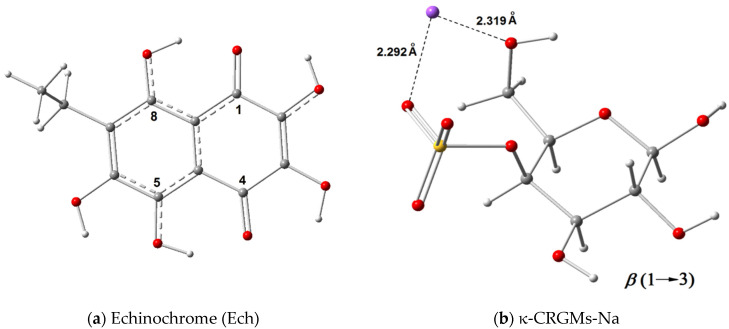
(**a**) Structure of Ech; (**b**) structure of monosaccharide—4-sulfat-Na-1,3-*β*-D-galactose of CRG.

The standardized substance Ech was used (Figure 3a). Ech is poorly soluble in water, but soluble in ethanol. We used an ethanolic solution of Ech (10 mg/mL) as a stock solution. Water solutions of CRG/Ech were obtained as described in the Methods section.

To study the binding between Ech and CRG, FTIR spectra of Ech and CRG/Ech were obtained (Figure 1). The absorption band ν(C=O) at 1572 cm^−1^ was observed in the FTIR spectrum of pure Ech, while another band at 1566 cm^−1^ was found in the in IR spectrum CRG/Ech (Figure 1b,c). The shift of about 6 cm^−1^ of the maximum absorption band to lower frequencies is probably due to the formation of complex between Ech and CRG. Moreover, the shift to a lower frequency in the complex CRG/Ech was observed to a band absorption at 1415 cm^−1^, whereas in the IR spectrum of pure Ech, the band absorption was observed at 1422 cm^−1^ (Figure 1b). Thus, dates of IR spectra showed that CRG interacts with Ech and forms a complex, which is consistent with data of UV spectra obtained earlier by us [18]. 

### 2.2. Quantum Chemical Calculations to Evaluate the Binding of Ech with CRG

In this work, quantum chemical calculations were used to prove the formation of the complex and determine the binding energy of the formed complex. 

For the evaluation of the enthalpy of complexes Ech with κ-CRG in calculation, we used monosaccharide 4-sulfat-1,3-*β*-D-galactose (κ-CRGMs-Na). Na^+^ cation forms two coordinate bonds in sulfated monosaccharide (Figure 3b).

Based on the calculations, it was shown that κ-CMS in complexes with Ech forms two coordinate bonds between Na^+^ cation and two oxygen atoms (Supplementary Figure S1) in the Ech molecule and one intermolecular hydrogen bond, −OH…O=S, between 2-*β*-hydroxyl group Ech and the sulfate group in κ-CMS. The number of coordinate bonds of Na^+^ cation in the complex is four. The total stabilization enthalpy of Ech-κ-CMS complexes varies about −Δ*H*_t_ = 5 − 8 kcal/mole depending on sites bonding 1′2, 2′3, 3′4, 4′5 and 5′6 (Table 1). In the complex of Ech with two κ-CMS, −Δ*H*_t_ is ~22, and with tree κ-CMS, it is ~30 kcal/mole. For the evaluation of the contributions of the electrostatic interaction to the total enthalpy of the complex Ech-κ-CMS, we used the Ech derivative 3O-methyl-Echinochrome A. In the complex 3OMe-Ech with κ-CMS, there are only four coordinate bonds between Na^+^ cation and four oxygen atoms: two belong to 3OMe-Ech, and the two other belong to κ-CMS (Supplementary Figure S2). The contribution of intermolecular hydrogen bonds to the total stabilization enthalpy of Ech-κ-CMS complexes is about 2.8 kcal/mole or 35%. The main contribution to total enthalpy is due to the coordinate bonds Na^+^ cation with oxygen atoms of the molecules of Ech (Table 2).

### 2.3. CRG/Ech Complex

Two samples of carrageenan isolated from the alga *C. armatus* were used in this work, namely gelling κ-CRG and unfractionated Σ-CRG, and their complexes with Ech were obtained. Complexes of Ech with Σ-CRG and κ-CRG were formed as described in the Methods. The ratio of the initial components CRG and Ech in the complexes was 10:1 (*v*/*v*). Thus, a 1 mL solution of CRG/Ech complex contained 10 mg of CRG and 1 mg of Ech. 

### 2.4. Liposome 

Liposomal drug delivery systems have long been successfully used in various fields of medicine for external and systemic use. In the present work, we included CRG/Ech in the liposomes and examined the antivirus properties of these liposomes.

**Figure 4:**
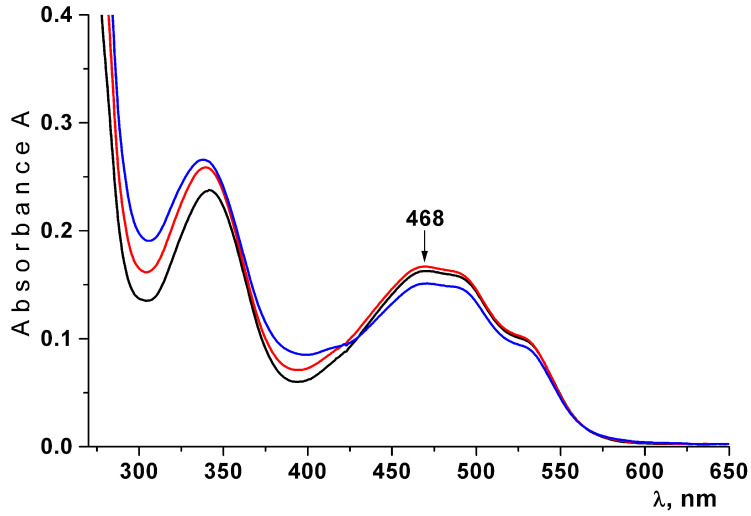
Absorption spectra of the Ech extracted with butanol from freshly prepared liposomes (black lines); from freshly prepared lyophilized dried liposomes (red line); from lyophilized dried liposomes after 30 days of storage at 4 after 30 days of being kept at 4 °C (blue line).

Water solutions of CRG/Ech were loaded into the liposome formulation by using a standard thin film method, followed by sonication. The effect of the lyophilization of liposomes on their stability was evaluated. The stability of Ech in the liposomes was determined by measuring the absorption value at 468 nm. The characteristic absorptions bands of the native Ech at 339 nm and 468 nm were observed in the absorption spectrum, indicating that Ech was not oxidized and remained stable after being encapsulated in liposomes (Figure 4). The lyophilization process did not violate the native form of Ech.

The SEM analysis revealed the formation of a population of heterogeneous vesicles ranging from 419.5 ± 12.0 to 494.8 ± 4.0 nm in size (Supplementary Figure S3).

### 2.5. Antiherpetic Activity of Tested Compounds

#### 2.5.1. Cytotoxicity and Anti-HSV-1 Activity of CRG/Ech Complexes 

The cytotoxicity assessment of the tested CRGs (κ-CRG and Σ-CRG), their complexes with Ech (κ-CRG/Ech and Σ-CRG/Ech), and reference compound (acyclovir) against Vero cells was determined by MTT assay. The investigated compounds had low cytotoxicity: their CC_50_ values were above 1000 µg/mL, while Ech showed a CC_50_ value of 142 µg/mL (Table 3).

To study the inhibitory effect of the tested compounds on the stages of HSV-1 infection, a plaque-reduction assay was performed. For that purpose, HSV-1 was pre-incubated with the compounds before infection (pretreatment of virus); cells were pre-incubated with the compounds before the viral challenge (pretreatment of cells); and compounds were added to cells during the adsorption of HSV-1 (simultaneous treatment) and after adsorption of virus to the cells (treatment of infected cells) (Table 3).

The pretreatment of HSV-1 with different concentrations (5–500 µg/mL) of the tested compounds (direct virucidal effect) showed that the CRGs/Ech complexes exhibited significantly higher antiviral activity compared with CRGs: the inhibitory concentration (IC_50_) of the κ-CRG/Ech complex was 20 times lower than that of the κ-CRG (3.7 μg/mL and 80 μg/mL, respectively), and the IC_50_ of the Σ-CRG/Ech complex was 7 times lower than that of the Σ-CRG (2.8 μg/mL and 20 μg/mL, respectively). At the same time, the difference between the IC_50_ values of CRGs/Ech complexes and Ech was inessential (*p* > 0.05). However, the selectivity indices (SI = CC_50_/IC_50_) of these compounds characterizing their efficacy and safety differed significantly (*p* ≤ 0.05): the SI of the κ-CRG/Ech and the Σ-CRG/Ech were 270 and 350, respectively, and the SI of the Ech, a more cytotoxic compound compared to CRGs and their complexes, was 35. It should be noted that the amount of Ech in the CRGs/Ech complexes was insignificant. Acyclovir, with this method of compound application, did not show antiviral activity (Table 3).

The treatment of Vero cells with compounds before infection (preventive effect) showed that κ-CRG reduced the HSV-1 plaque formation most effectively (SI 111), Ech was the least active (SI 1.7), and acyclovir had no preventive effect. At the same time, CRGs/Ech complexes (κ-CRG/Ech and Σ-CRG/Ech) protected Vero cells against herpesvirus infection, with SI values 1.8–2.8 times lower than the SI values of κ-CRG and the Σ-CRG, respectively (*p* ≤ 0.05) (Table 3). Apparently, the observed preventive effect of the investigated carrageenan complexes with Ech is due to the action of CRGs, while the decrease in the ability of CRGs/Ech complexes to protect cells from the HSV-1-induced cytopathic effect can be explained by the toxicity of Ech.

Next, the tested compounds and HSV-1 were added to Vero cells simultaneously to investigate their influence on the early stages of the virus–cell interaction. In this assay, the CRGs/Ech complexes exhibited higher inhibitory activity against HSV-1 compared with CRGs: the IC_50_ values of the κ-CRG/Ech and Σ-CRG/Ech were 4.7–3.5 times lower than the IC_50_ values of the κ-CRG and Σ-CRG, and the SI values of the complexes were 2.3–1.8 higher than the SI values of the CRGs, respectively (*p* ≤ 0.05) (Table 3). Meanwhile, the SI of the Ech (4.1) was much lower than that of the CRGs and their complexes, whereas acyclovir displayed the highest antiherpetic activity (SI > 950).

The application of CRGs after virus adsorption and penetration to cells (at 1 h postinfection) showed a moderate inhibition of HSV-1 replication (the average SI was 20) and a modest virus-inhibiting activity of CRGs/Ech complexes against this virus (SI ~10), wherein the difference between the IC_50_ values of CRGs and CRGs/Ech complexes was not significant (the average in their IC_50_ was 90 µg/mL). The postinfection treatment of cells with Ech had a weak effect on virus replication in contrast to acyclovir (SI of <1.5 vs. 20,000) (Table 3).

#### 2.5.2. Anti-HSV-1 Activity of the Liposomal Form of the Carrageenan Complex (Σ-CRG/Ech) 

The effect of the liposomal form of the Σ-CRG/Ech complex on different stages of the HSV-1 infection was studied. This complex was chosen for inclusion into liposomes because it demonstrated antiviral activity by affecting the early stages of the HSV-1 lifecycle, especially the virus, directly.

The cytotoxicity of empty and complex-loaded liposomes against Vero cells was evaluated before testing their antiviral activity. The results of the MTT assay showed that both the liposomal form of the Σ-CRG/Ech complex and empty liposomes had low toxicity to cells: their CC_50_ was above 2000 µg/mL (Supplementary Table S1). 

**Figure 5:**
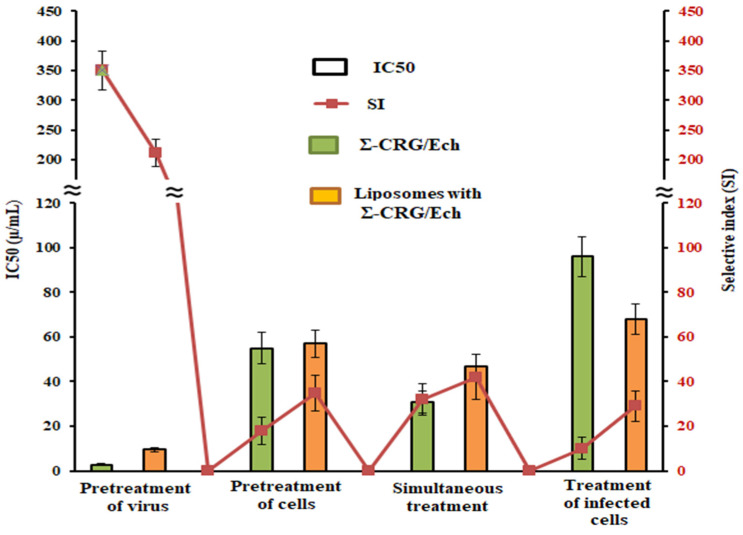
The anti-HSV-1 activity of the Σ-CRG/Ech complex and its liposomal form at the different treatment schemes. The antiviral activity of the compounds was determined by the plaque reduction assay and was expressed as a 50% inhibitory concentration, IC_50_ (columns), and a selective index, SI (line). Σ-CRG/Ech, carrageenan complex with echinochrome; liposomes with Σ-CRG/Ech, liposomal form of the complex. The results were obtained by applying compounds at different stages of the HSV-1 infection, and each data point represents the mean value ± SD of three independent experiments.

The anti-HSV-1 activity of the liposomal form of the Σ-CRG/Ech complex was assessed by using a plaque-reduction assay. The results of the study are presented in Figure 5 and in Supplementary Table S1. A comparative analysis of the antiviral activity of the complex and its liposomal form showed that the Σ-CRG/Ech complex had higher virucidal activity (directly acting on the virus) than its liposomal form: IC_50_ (2.8 and 9.5) and SI (350 and 211), respectively (*p* ≤ 0.05). At the same time, the study of the preventive effect (directly acting on the Vero cells) of the tested compounds revealed a higher level of prophylactic activity of the liposomal form (SI 35) compared to the Σ-CRG/Ech complex (SI 18), and this may be associated with the lower toxicity of the liposomal form (Figure 5). Moreover, the simultaneous treatment of cells with the tested compounds and virus displayed a sufficiently high antiviral activity of the liposomal form of the complex (SI 42), although this method showed no significant difference between Σ-CRG/Ech complex and its liposomal form (*p* > 0.05). It should be noted that the application of the liposomal form of Σ-CRG/Ech complex after virus adsorption and penetration to cells (at 1 h postinfection) effectively reduced the HSV-1 plaque formation: its virus-inhibiting activity was three times higher than that of the Σ-CRG/Ech complex (SI 29 and SI 10, respectively) (*p* ≤ 0.05). Empty liposomes showed no activity in the above experiments.

## 3. Discussion

Viral infections remain a major threat to humans and animals, and there is a crucial need for new antiviral agents as the risk of developing antiviral resistance increases [28,29]. It is known that CRG and its low-molecular-weight derivatives show a good effect in the prevention of a wide range of diseases, mainly caused by enveloped viruses. CRGs might inhibit viral infection via direct actions on the virus surface by its negative charge [6,8,30]. Carrageenan-based vaginal mucoadhesive solid formulations have been used for the controlled release of a well-known antiherpetic drug, acyclovir, for the prevention of sexual herpes virus infection [31].

Previously, we tested the effect of CRGs isolated from a different family of red algae of Pacific Coast at the stage of viral infection and showed the dependence of antiviral activity on the structural features of these polysaccharides [13]. According to the data obtained by us, CRGs containing DA-units (3.6-anhydrogalactos) inhibited the binding of the virus to cells significantly and exhibited the high anti-HSV-1 activity at the stage of virus attachment to cells significantly more compared with other CRGs. In these polysaccharides (with the κ-carrabiose units), DA adopts the 1C4-chair conformation that is crucial for the formation a three-dimensional network structure that inhibits the adsorption of the virus, thus providing the greatest virus-inhibiting effect or attachment [32,33]. It should be noted that these CRGs showed an insufficiently strong virucidal effect. However, on the other hand, as our data showed, the polysaccharide-containing mixture of two types of CRGs, kappa and lambda, exhibited higher virucidal activity compared to the other CRGs [13]. This is probably due to the fact that lambda CRG has the conformation of a chaotic coil, which ensures their flexibility and to interact more effectively with some glycoproteins of the viral envelope, which are necessary for the adsorption of HSV-1 to cells. Moreover, this CRG contains more sulphate group than other CRGs. On the basis of the data obtained earlier, in the presented work, we used the most active samples of CRGs: Σ-CRG (the mixture of kappa with lambda) and pure kappa CRG in combination with Ech to study their anti-herpetic action.

As previously shown, Ech exerts significant anti-HSV-1 activity, mainly due to its direct virucidal properties [19,20]. Ech also showed a moderate inhibitory effect at the early stage of a viral infection, when the test compound simultaneously affects both HSV-1 and Vero cells, but it did not suppress HSV-1 replication when it treated infected cells (virus-inhibiting effect). At the same time, Ech slightly inhibited the attachment of the virus to the cell and, to a lesser extent, the penetration of the virus into the cell. 

Taking into account the different effects of CRGs and Ech at the stage of viral infection, we obtained CRG/Ech complexes in order to enhance the virucidal and preventive action of these marine substances. The main disadvantage of Ech is its high cytotoxicity. The inclusion of a low dose of Ech in the complex with CRG makes it possible to reduce its toxicity and, at the same time, maintain or increase the antiviral activity of the CRG/Ech complex.

Based on quantum chemical calculations obtained in this work, it was shown that CRG forms stable complexes with Ech. During the formation of complexes, the main contribution is made by the electrostatic interaction of the sodium cation with the oxygen atoms of the Ech molecule. 

The study of the effect of the obtained CRG/Ech complexes on different stages of HSV-1 infection showed the ability of both complexes to directly affect virus particles (virucidal effect), to increase the resistance to virus infection (preventive effect), and to inhibit virus–cell interactions (virus-inhibiting effect). It should be noted that, in such complexes (CRG:Ech 10:1 *w*/*w*), only one part of Ech falls on 10 weight parts of CRG. The tested complexes exerted the greatest antiviral activity during the pretreatment of the virus with these compounds. The calculated selective indices testified to the increased safety and efficacy of the proposed compositions compared to Ech. With preventive action, CRGs/Ech complexes protected Vero cells from the cytopathic effect of HSV-1 higher than the Ech but less than for the CRGs themselves. In addition, both carrageenan complexes (κ-CRG/Ech and Σ-CRG/Ech) showed a higher inhibitory effect of blocking virus-cell interactions compared to CRGs. We believe that the high virucidal and anti-adsorption activities of the CRGs/Ech complexes against HSV-1may be a result of multivalent interactions of the tested compounds with both the virus and its cellular receptors.

Liposomes are microscopic vesicle composed of phospholipid bilayers that have attracted a lot of interest as pharmaceutical carriers. The liposomal coated with Σ-CRG/Ech complex was evaluated in this work for its antiherpetic activity. This liposomal formulation was prepared based on conventional liposome. The study of the anti-HSV-1 activity of the liposomal form of the Σ-CRG/Ech complex showed that, compared with the Σ-CRG/Ech complex, its liposomal form has less toxicity against Vero cells, more effectively protects cells from the HSV-1-induced cytopathic effect, and more effectively inhibits the early stage of viral replication.

## 4. Materials and Methods

### 4.1. Virus and Cell Culture

In this study, HSV-1 strain L2 was obtained from the N.F. Gamaleya Federal Research Centre for Epidemiology and Microbiology, Moscow, Russia. Determination of the cytotoxicity and antiviral activity of the compounds was carried out on a Vero cell culture (kidney epithelial cells of the African green monkey Chlorocebus sp.) obtained from the N.F. Gamaleya Federal Research Centre for Epidemiology and Microbiology, Moscow, Russia. HSV-1 was grown in Vero cells, using Dulbecco’s Modified Eagle’s Medium (DMEM, Biolot, St. Petersburg, Russia) supplemented with 10% fetal bovine serum (FBS, Biolot, St. Petersburg, Russia) and 100 U/mL of gentamycin (Dalkhimpharm, Khabarovsk, Russia), at 37 °C in a CO_2_ incubator. In the maintenance medium, the FBS concentration decreased to 1%. 

### 4.2. Extraction of Carrageenans (CRGs) 

The red algae *Chondrus armatus* (Gigartinaceae were harvested at the end of August in The Peter the Great Bay Japan Sea and identified by Prof. E. Titlynov and T. Titlynova (National Scientific Centre of Marine Biology, Far-Eastern Branch of the Russian Academy of Sciences). The selected seaweeds were in the vegetative form, lacking any reproductive organs. The algae were treated with acetone to remove pigment. Dried and milled algae (50 g) were suspended in hot water (1.5 L), and the polysaccharides were extracted at 90 °C for 2 h in a water bath. The residue was removed by centrifugation, and supernatant was poured into ethanol (three volumes), yielding the crude unfractionated form of polysaccharides. The crude polysaccharides were purified by redissolving in water, filtered through a Vivaflow200 membrane (Sartorius, Göttingen, Germany) with a pore size of 100 kDa, concentrated, dialyzed, and lyophilized, yielding total polysaccharide Σ-CRG. Then the Σ-CRG was separated into gelling KCl-insoluble and non-gelling KC1-soluble fractions, as described previously [26], and their structures were established according to the published protocol by methods spectroscopy.

### 4.3. Analytical Methods 

To determine the content of 3,6-anhydrogalactose, total reductive hydrolysis of the CRGs in 2 M Trifluoroacetic acid (TFA) (100 °C, 4 h) with 4-methylmorpholinborane was carried out, and then aldononitrile acetates were obtained [34]. 

### 4.4. Spectroscopy Methods 

IR spectra of the polysaccharides (as films) were recorded on a Fourier-transform spectrophotometer INVENIO S (Bruker, Billerica, MA, USA), taking 120 scans with 4 cm^–1^ resolution. The spectra regions of 1900–700 cm^−1^ were used, and the baseline was corrected for scattering. The spectra were normalized by the monosaccharide ring skeleton absorption at 1074 cm^–1^ (A_1074_ ≈ 1.0). Absorption spectra were recorded by spectrophotometer Shimadzu 3600 (Japan). ^13^C Nuclear magnetic resonance (NMR) spectra were recorded by using a DRX-500 (125.75 MHz) spectrometer (Bruker, Hamburg, Germany) operating at 50 °C. The polysaccharides (3 mg) were deuterium-exchanged twice with heavy water (D_2_O, 0.6 mL) by freeze-drying prior to examination in a solution of 99.95% D_2_O. Chemical shifts were described relative to the internal standard, acetone (δC 31.45, δH 2.25). The NMR data were acquired and processed by using XWIN-NMR 1.2 software (Bruker). 

### 4.5. Molecular Weight Determination 

The molecular masses of polysaccharides were calculated by the Mark–Howink equation: [η] = K × Ma, where [η] is the intrinsic viscosity, and K and α are empirical constants for CRG [35] and CH [36]. For this, the viscosity of CRG and CH solutions was measured (0.1–1.0 mg/mL in 0.1 M NaCl and 2.0–10.0 mg/mL in 0.2 M NaCl/0.2 M AcOH, respectively) on a modified Ubbelode viscometer (OKB Pushchino, Russia) with a capillary diameter of 0.3 mm, at 25 °C; the timing accuracy was ± 0.1 s. The intrinsic viscosity of the samples was calculated by extrapolating the ln (η) × C ^−1^ dependence to infinite dilution, using the least-squares method, and viscosimetric molecular weights were calculated.

### 4.6. Quantum Chemical Calculations

All quantum chemical calculations were performed by using the method PCM-B3LYP/6-31 + (d, p) in water, using program complex Gaussian 16W^1^ (Gaussian 16 W, Version 1.1, Gaussian Inc., Wallingford, CT, USA, (2019)). Localization of stationary points on the potential energy surface (PES) was carried out with full optimization of all geometric parameters. In the absence of imaginary frequencies, stationary PES points were referred to as energy minima. The free-energy Gibbs and enthalpy of complexes Ech with κ-CRGMs were calculated by using Equation (1):

Δ*G*/Δ*H* = *G*(complex)/*H*(complex) − Σ*G*(reactants)/Σ*H*(reactants), (1)

where reactants are κ-CRGMs and Ech or 3OMe-Ech.

### 4.7. Preparation of CRG/Ech Complex 

The standardized substance Echinochrome (pentahydroxyethylnaphthoquinone) in powder form (registration number in the Russian Federation is P N002362/01) [Russian State Register of Drugs (December 2016) Part 2] was obtained in G.B. Elyakov Pacific Institute of Bioorganic Chemistry, FEB of the Russian Academy of Sciences*,* Vladivostok. A solution of 1% CRG was prepared by dissolving 10 mg CRG in 1 mL deionized water at 50 °C, while stirring on a stirrer. Stock solution of Ech in a concentration of 10 mg/mL was prepared. To obtain a CRG/Ech complex, 0.1 mL of a stock solution of Ech was added to 1 mL of a 1% CRG solution. The mixture was left under stirring in a dark place at a temperature of 37 °C for 60 min. Thus, the solution of CRG/Ech complex was obtained at a ratio of the initial components, 10:1 (*w*/*w*).

The concentration of Ech in the solution was measured by using absorption spectra at λ = 468 nm.

### 4.8. Preparation of Liposomes

Conventional liposomes were obtained by using standard thin film hydration. Egg lecithin (10% in 1.6 mL) and cholesterol (69.9 mg/mL) solutions were prepared in chloroform–methanol 9:1 (*v*/*v*) and mixed. The mixture was precipitated as a film on the wall of a glass test tube by evaporating the solvent with a vacuum-line. The test tubes were then placed under a vacuum for at least 2 h to remove residual solvent. The thin film of the lipid was hydrated with 1 mL H_2_O, using the sonication, and then characterized. An extruder with membranes of 0.4 µm was used for obtaining more homogeneous samples. 

CRG/Ech-containing liposomes were produced by performing the next steps. At first, the complex CRG/Ech (10:1 *w*/*w*) was prepared as described above. Then 1 mL of this solution was added to the dried lipid film. The mixture was sonicated three times for 15 min in an ultrasonic bath, and then 0.1 mL suspension was added to 1.4 mL H_2_O in different tubes and then centrifuged for 15 min at 15,000× *g*. The supernatant was removed, and the pellet was suspended in 1 mL of water and again centrifuged. The centrifugation step was carried out to remove the CRG/Ech complex not included in the liposomes. Washing was performed twice. An extruder was used to reduce the heterogeneity of the liposomes. The suspension of liposomes was passed through 0.4-micron membranes 10 times, on an extruder, according to the instructions. 

### 4.9. Virological Methods

For cytotoxicity and antiviral-activity determination, the tested compounds—different structural types of CRGs, complexes of CRGs with Ech and the liposomal form of the carrageenan complex (Σ-CRG/Ech)—were diluted in DMEM. Echinochrome A (98.0%, pharmaceutical, state registration number PN002362/01-2003, G.B. Elyakov Pacific Institute of Bioorganic Chemistry FEB RAS, Russia, Vladivostok) was dissolved in dimethyl sulfoxide (DMSO, Sigma, Saint-Louis, MO, USA) and stored at −20 °C. For the experiments, a stock solution (10 mg/mL) of it was diluted with DMEM to a final concentration of 0.5% DMSO. Acyclovir^®^, freeze-dried powder for injections (GlaxoSmithKline Pharmaceuticals S.A., Poznan, Poland), was used as a reference compound and diluted in DMEM.

#### 4.9.1. Cytotoxicity of the Tested Compounds 

The cytotoxicity evaluation of the studied carrageenans was performed by using the MTT assay, as previously described [13]. In brief, confluent Vero cells (1 × 10^4^ cells/well) in 96-well microplates were incubated with various concentrations of the tested compounds (1–2000 µg/mL) at 37 °C for 72 h (5% CO_2_); untreated cells were used as controls. MTT solution (methylthiazolyltetrazolium bromide, Sigma, St. Louis, MO, USA) was added to cells at a concentration of 5 mg/mL, following incubation for 2 h at 37 °C. Then the MTT solution was removed, and isopropanol was added to dissolve the insoluble formazan crystals. The optical density was read at 540 nm (Labsystems Multiskan RC, Vantaa, Finland). Cytotoxicity was expressed as the 50% cytotoxic concentration (CC_50_) of the tested compound that reduced the viability of treated cells by 50% compared with control cells [37]. Experiments were performed in triplicate and repeated three times. 

#### 4.9.2. Anti-HSV-1 Activity of the Tested Compounds 

The inhibitory effects of the tested compounds on HSV-1 replication cycle stages in Vero cells were evaluated by the plaque reduction assay [38]. Vero cell monolayers grown in 24-well plates (1 × 10^5^ cells/well) were infected with 100 PFU/mL of HSV-1. Several of the tested compounds and Acyclovir^®^ application schemes were investigated; each was performed in three independent replicates, with different concentrations of compounds (5–500 µg/mL). The plates were incubated for 72 h at 37 °C (5% CO_2_) until plaques formed. These schemes were as follows:

Pretreatment of virus with compounds: The virus was mixed with compounds at a 1:1 (*v*/*v*) ratio and preincubated for 1 h at 37 °C. Then the mixture was applied to the cellular monolayer and incubated for 1 h at 37 °C. After removing the virus–compound mixture, the cells were washed with phosphate-buffered saline (PBS), covered with the maintenance medium (DMEM) containing 1% carboxymethyl cellulose (CMC, ICN Biomedicals Inc., Aurora, OH, USA), and incubated for 72 h at 37 °C (5% CO_2_).

Pretreatment of cells with compounds: A monolayer of cells was pretreated with compounds for 1 h at 37 °C before infection. The cells were washed with PBS to remove the compounds and infected with the virus for 1 h at 37 °C. Then unabsorbed virus was removed by washing with PBS, and the cells were incubated with DMEM with 1% CMC.

Simultaneous treatment of the cells with the tested compounds and virus: The monolayer of cells was infected with the virus and simultaneously treated by the compounds (virus: compound, 1:1 *v*/*v*) for 1 h at 37 °C. After virus adsorption, the virus–compound mixture was removed; the cells were washed with PBS and incubated in maintenance medium until CPE appeared.

Treatment of infected cells: The monolayer of cells was infected with the virus at 37 °C for 1 h, and then it was washed and covered with DMEM with 1% CMC containing different concentrations of the tested compounds.

In all assays, after 72 h of incubation, the cells were fixed with cold ethanol for 20 min, stained with a solution of 0.5% crystal violet in 20% ethanol, and the viral plaques were then counted. The plaque formation inhibition rate was calculated according to the following formula [39]: plaque inhibition (%) = 100 − [(PT/PC) × 100], where PT and PC are the plaque number of compound-treated infected cells and the virus control (without compound), respectively. The IC_50_ of each compound was determined as the compound concentration that inhibited 50% of viral plaque formation, compared to the control. The SI was calculated as the ratio of CC_50_ to IC_50_ for each compound. Experiments were repeated three times.

### 4.10. Statistical Analysis

Statistical processing of the data was performed by using the Statistica 10.0 software (StatSoftInc., Tulsa, OK, USA). CC_50_ and IC_50_ were calculated by regression analysis of the dose–response curves. The results are presented as the mean ± standard deviation (SD). The differences between the parameters of the control and experimental groups were estimated by using the Wilcoxon test. Differences were considered significant at *p* ≤ 0.05.

## 5. Conclusions

In the present study, we evaluated the antiherpetic activity of carrageenan complexes with echinochrome and its liposomal form. By quantum chemical calculations, it was shown that CRG forms stable complexes with Ech. The results of our study showed that complexes of kappa CRG and Σ-CRG with Ech exhibit significant anti-HSV-1 activity, mainly due to virucidal and prophylactic properties, as well as their ability to inhibit virus–cell interaction. The liposomal form of the Σ-CRG/Ech complex after virus adsorption and penetration to cells effectively reduced the HSV-1 plaque formation. The virus-inhibiting activity of the liposomal form of the Σ-CRG/Ech complex was three times higher than that of the Σ-CRG/Ech complex itself. Obtaining CRGs/Ech complexes and their liposomal forms can become the basis of a successful strategy for the development of promising antiherpetic drugs.

## Figures and Tables

**Table 1:** Characteristics of CRGs from *Chondrus armatus*.

Sample of CRGs	Structure of Disaccharide Repeating Unit		Content, Dray Weight %			MW, kDa
	3-Linked	4-Linked	Gal	3,6 AnGal	SO_4_^−2^	
κ-CRG	G4S	DA	37.1	33.0	22.0	560
Σ-CRG κ + λ	G4S G2S	DA D2S, 6S	40.1	16.2	27.1	185

Note: G: 1,3-β-D-galactose; DA: 1,4-3,6-anhydro-α-D-galactose; G4S: 1,3-β-D-galactose 4-sulfate; DA2S: 1,4-3,6-anhydro-α-D-galactose 2-sulfate. Gal: galactose; 3,6-AnGal: 3,6-angidrogalactose; SO_4_^2−^: sulphate group.

**Table 2:** Enthalpy (Δ*H*) and Gibbs energy (Δ*G*) of complexes of Ech with κ -CRG.

Sample	−Δ*H*, kcal/mole	−Δ*G*, kcal/mole
κ -CRG-Na-1′2 *-Ech	8.27	2.42
κ -CRG-Na-2′3-Ech	4.90	6.31
κ -CRG-Na-3′4-Ech	7.91	3.44
κ -CRG-Na-3′4-3OMe-Ech	5.14	9.25
κ -CRG-Na-5′6-Ech	7.20	3.78
2- κ -CRG-Na-1′2-3′4-Ech	21.63	1.95
3- κ -CRG-Na-1′2-3′4-5′6-Ech	29.77	6.20

* Number of sites bonding Na^+^ cation with oxygen atoms of molecules Ech.

**Table 3:** Antiherpetic activity of complexes of different structural types of carrageenans with echinochrome.

Compounds	CC_50_	Pretreatment of Virus		Pretreatment of Cells		Simultaneous Treatment		Treatment of Infected Cells	
		IC_50_ (µ/mL)	SI	IC_50_ (µ/mL)	SI	IC_50_ (µ/mL)	SI	IC_50_ (µ/mL)	SI
Ech	142 ± 6	4.1 ± 0.6	35 ± 5	83 ± 14	1.7 ± 0.2	35 ± 6	4.1 ± 0.5	95 ± 18	1.5 ± 0.2
κ-CRG	>2000	80 ± 10	25 ± 3	18 ± 3	111 ± 14	56 ± 8	36 ± 5	77 ± 11	26 ± 4
Σ-CRG	>2000	20 ± 4	100 ± 5	60 ± 7	33 ± 4	109 ± 16	18 ± 2	103 ±14	19 ± 3
κ-CRG/Ech	>1000	3.7 ± 0.5 *	270 ± 38 *	25 ± 4	40 ± 6 *	12 ± 2 *	83 ± 12 *	80 ± 12	12 ± 2 *
Σ-CRG/Ech	>1000	2.8 ± 0.4 *	350 ± 54 *	55 ± 7	18 ± 3 *	31 ± 5 *	32 ± 5 *	96 ± 14	10 ± 2 *
ACV	>1000	NA		NA		2.1 ± 0.3	>950	0.1 ± 0.01	>20.000

Note: Values represent the means ± standard deviations of three or more independent experiments; κ-CRG and Σ-CRG—various structural types of carrageenans. κ-CRG/Ech and Σ-CRG/Ech—complexes of various types of carrageenans with echinochrome A. Acyclovir (ACV) and echinochrome A (Ech) were used as reference compound; IC_50_, concentration that inhibited 50% of viral plaque formation; SI, selectivity index (CC_50/_IC_50_); NA—no activity. * Significance of the differences between the parameters of carrageenan polysaccharides (κ-CRG and Σ-CRG) compared to the corresponding complex of carrageenan with echinochrome A (κ-CRG/Ech and Σ-CRG/Ech) (*p* ≤ 0.05).

## Supplementary Materials

Supporting information can be downloaded at: https://www.mdpi.com/article/10.3390/ijms232415754/s1, Table S1: Antiherpetic activity of the liposomal form of Σ-CRG/Ech. Figure S1. PCM-B3LYP/6-31+G(d,p) optimized geometries of the most stable complex κ-CRGS with Ech at positions C(1)=O and C(2)–OH («k-CRG-Na-1′2-Ech»). Figure S2. PCM-B3LYP/6-31+G(d,p) optimized geometries of the complex κ-CMS with 3-OMe-Ech at positiones C(3)–O and C(4)=O («k-Car-Na-1′2-Ech») «k-Car-Na-3′4-3OMe-Ech». Figure S3. Scanning electron microscopy images of CRG/Ech-containing liposomes. Mag. = 50.87 KX, EHT = 10.00 kV.
