# Supplementary material for: Antiherpetic Activity of Carrageenan Complex with Echinochrome A and Its Liposomal Form
Source: Int J Mol Sci. 2022 Dec 12;23(24):15754. doi: 10.3390/ijms232415754 (PMC9779482; doi:10.3390/ijms232415754)
Supplement: Supplementary file 1 [file ijms-23-15754-s001.zip › ijms-2081290-supplementary.pdf]

Supplementary Materials:

Table S1. Antiherpetic activity of the liposomal form of Σ-CRG/Ech

Compounds	CC_{50}	Pretreatment of virus		Pretreatment of cells		Simultaneous treatment		Treatment of infected cells	
		IC_{50} (μ/mL)	SI	IC_{50} (μ/mL)	SI	IC_{50} (μ/mL)	SI	IC_{50} (μ/mL)	SI
**Liposomes Σ-CRG/Ech**	>2000	9.5 ± 1,1*	211 ± 23*	57 ± 7	35 ± 5*	47 ± 6	42 ± 6	68 ± 9*	29 ± 4*
**Σ-CRG/Ech**	>1000	2.8 ± 0.4	350 ± 54	55 ± 7	18 ± 3	31 ± 5	32 ± 5	96 ± 14	10 ± 2
**Empty liposomes**	>2000	NA		NA		NA		NA	

Note: Values represent the means ± standard deviations of three or more independent experiments; Σ-CRG/Ech - complex of carrageenan Σ-CRG with echinochrome A; Liposomes Σ-CRG/Ech - liposomal form of this complex; IC_{50}, concentration that inhibited 50% of viral plaque formation; SI, selectivity index (CC_{50} /IC_{50}); NA - no activity. * Significance of the differences between the parameters of complex of carrageenan Σ-CRG/Ech compared to the liposomal form of this complex ($p \leq 0.05$)

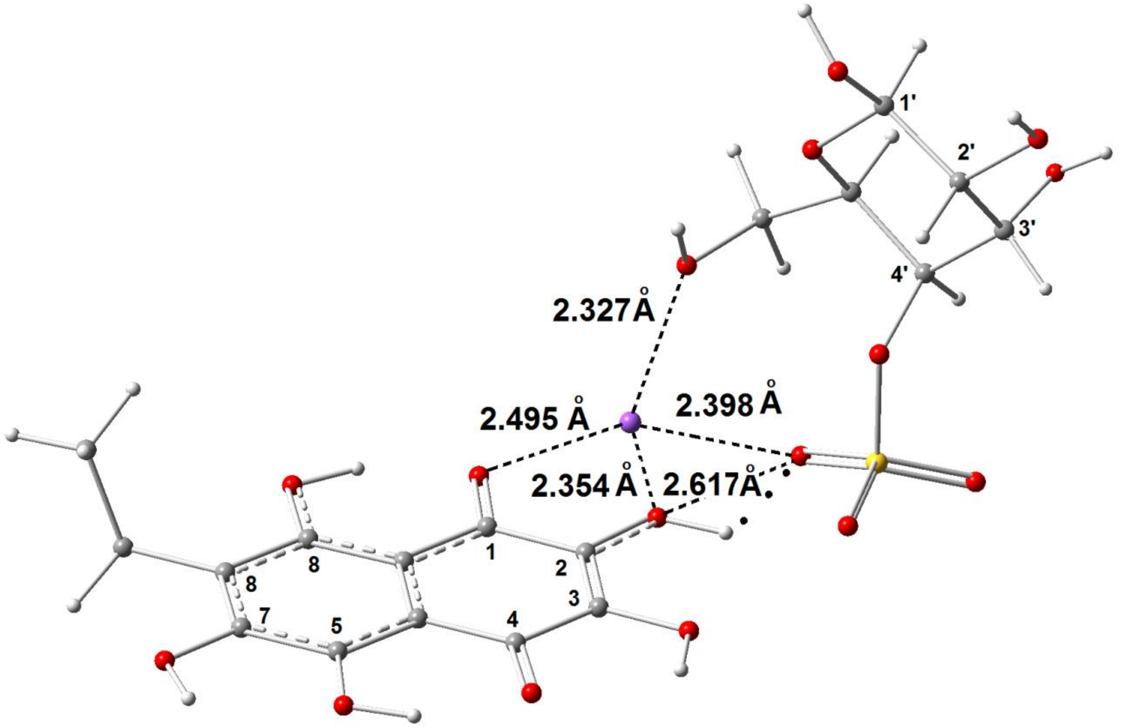

Figure S1. PCM-B3LYP/6-31+G(d,p) optimized geometries of the most stable complex κ-CRGS with Ech at positions C(1)=O and C(2)–OH («k-CRG-Na-1′2-Ech»)

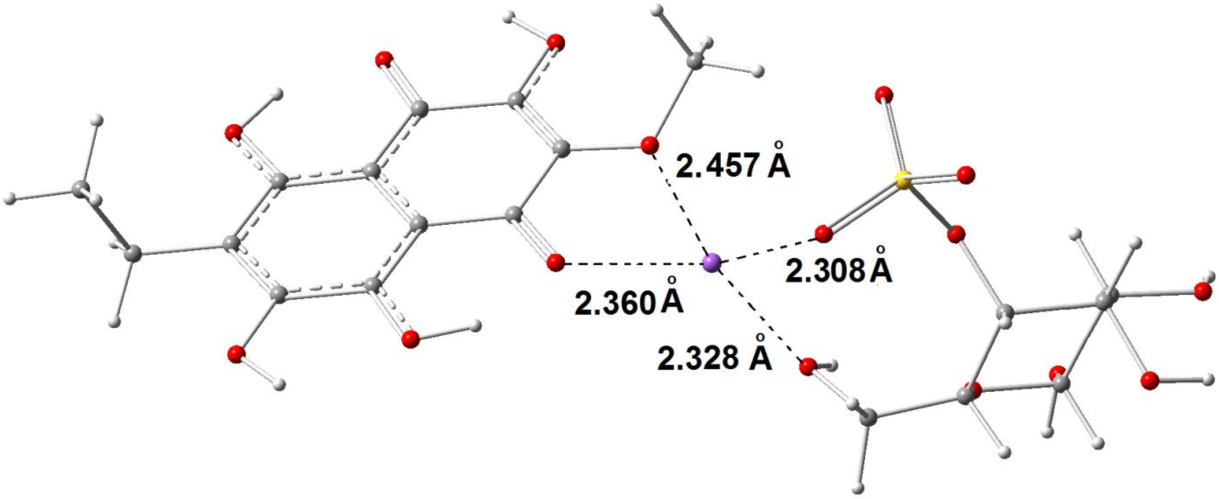

Figure S2. PCM-B3LYP/6-31+G(d,p) optimized geometries of the complex κ-CMS with 3-OMe-Ech at positiones C(3)–O and C(4)=O («k-Car-Na-1′2-Ech») «k-Car-Na-3′4-3OMe-Ech»

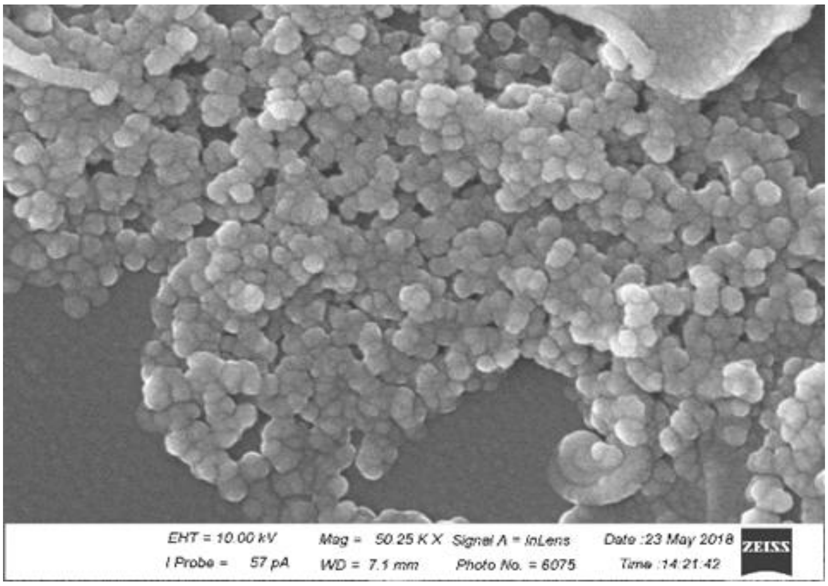

Figure S3. Scanning electron microscopy images of CRG/Ech-containing liposomes. Mag. = 50.87 KX, EHT = 10.00 kV
